# Association between Neck Circumference and the Risk of Decreased Estimated Glomerular Filtration Rate in the General Population of China: A Cross-Sectional Study

**DOI:** 10.1155/2020/3496328

**Published:** 2020-11-23

**Authors:** Jing Xue, Bing Li, Jie Wang, Songyan Yu, Anping Wang, Ping An, Yiming Mu

**Affiliations:** ^1^Medical School of Chinese PLA, Beijing, China; ^2^Department of Endocrinology, The First Medical Center of Chinese PLA General Hospital, Beijing, China

## Abstract

**Background:**

The burden of chronic kidney disease is increasing substantially worldwide. Neck circumference (NC), an anthropometric index for upper-body adiposity, has been recognized as an indicator of cardiometabolic diseases. However, the association between NC and renal dysfunction has not been fully disclosed.

**Objectives:**

The aim of this study was to investigate the association between NC and estimated glomerular filtration rate (eGFR) in the general population in China.

**Methods:**

A total of 8805 participants (3322 men and 5483 women) were enrolled in this study. Logistic regression analysis was conducted to examine the association between NC and eGFR. The male and female subjects were divided into four groups according to the NC quartiles. The primary outcome was defined as an eGFR ≤ 90 mL/min/1.73 m^2^.

**Results:**

Logistic regression analysis revealed that in both sexes, higher NC was significantly associated with a higher risk of decreased eGFR even after fully adjusting for age, other anthropometric indexes, traditional risk factors for chronic kidney diseases, and sociologic risk factors (quartile 1 as a reference; the odds ratios [95% confidence intervals] were as follows: quartile 2, 1.26 [0.99-1.59]; quartile 3, 1.40 [1.07-1.83]; and quartile 4, 1.71 [1.22-2.38], in men; quartile 2, 1.14 [0.95-1.37]; quartile 3, 1.31 [1.03-1.66]; and quartile 4, 1.32 [1.04-1.68], in women). Stratified analyses suggested that the association was significant among subjects with prediabetes or normal serum uric acid levels and those without cardiovascular diseases (CVD). Among subjects with CVD, the association persisted only in males. When the subjects were stratified according to blood pressure or BMI, the association persisted among male subjects with abnormal blood pressure and was strengthened among male subjects who were overweight/obese, while the association disappeared among female subjects.

**Conclusions:**

NC is independently associated with the risk of decreased eGFR in the general population in China, indicating that NC could contribute to renal dysfunction risk assessment.

## 1. Introduction

Chronic kidney disease (CKD) is a critical public health problem worldwide and contributes to a large proportion of the medical burden in both developed and developing countries. Data from a recent study showed that the rank of CKD among the leading causes of death has risen continuously over the past 27 years globally [1]. With the increasing morbidity of obesity and obesity-related cardiometabolic diseases, the prevalence of CKD in China has also increased markedly, reaching 10.8% in 2010 [2]. Despite the high prevalence and mortality of CKD, the public's knowledge and awareness of CKD are low. Therefore, the identification of simple and reliable renal dysfunction-related indicators is of great importance.

In recent years, neck circumference (NC) has been recognized as a valid and simple anthropometric measure of upper-body fat distribution and a novel indicator of visceral fat accumulation [3–5] and cardiometabolic diseases [4, 6–11], which are leading causes of CKD. Preis and his colleagues discovered a significant relationship between NC and type 2 diabetes mellitus (T2DM) even after fully adjusting for confounding factors [6]. Vallianou et al. found that NC was positively associated with triglycerides (TGs) but negatively associated with high-density lipoprotein cholesterol (HDL) [8]. A variety of studies have also demonstrated that NC is independently associated with hypertension [12, 13] and hyperuricemia [9] in individuals with diverse ethnic backgrounds. Interestingly, some studies have demonstrated that compared with traditional anthropometric indexes such as body mass index (BMI) and waist circumference (WC), NC was a better indicator of postprandial hyperglycemia, prediabetes, and hyperlipidemia [14, 15], implying the extensive application of NC in obesity-related diseases. Furthermore, NC is stable and rarely affected by respiration or diet and is especially suitable for people with disabilities or bedridden individuals for whom it was infeasible to measure BMI or WC accurately.

Although NC is a valid indicator of obesity and cardiometabolic diseases, which are closely related to CKD, direct research on the relationship between NC and renal dysfunction is very limited. One study including 177 patients in the Department of Cardiology found that NC was a predictor of renal insufficiency in these high-cardiovascular-risk patients [16]. However, this small sample study did not fully adjust for confounding factors and targeted specific populations, limiting its application in the general population. Therefore, this cross-sectional study is based on a large sample of the general population in China and is aimed at exploring the relationship between NC and estimated glomerular filtration rate (eGFR), a widely used index to identify renal dysfunction.

## 2. Materials and Methods

### 2.1. Study Population

**Figure 1:**
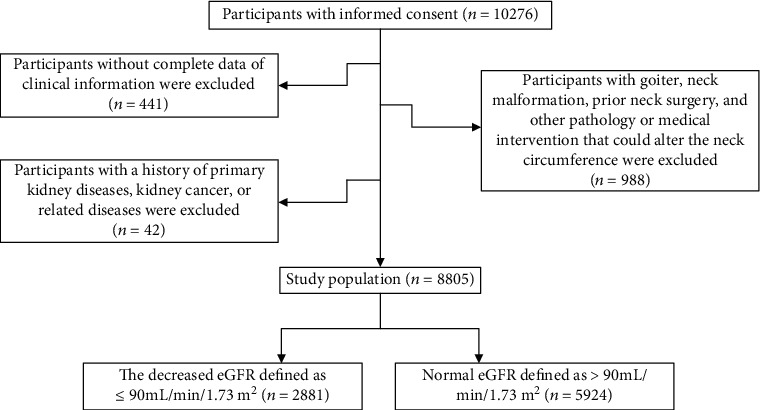
Selection of the study population.

The REACTION (Risk Evaluation of cAncers in Chinese diabeTic Individuals) study is an ongoing multicenter prospective cohort study. The REACTION study is aimed at obtaining a comprehensive understanding of the association between T2DM and cancer in China, as reported previously [17]. Our study population was from the Beijing subcenter of the REACTION study. Between April and October 2015, a total of 10276 individuals aged over 40 were recruited. After excluding participants who met the exclusion criteria (Figure 1), 8205 participants were enrolled.

### 2.2. Data Collection

All participants received comprehensive examinations, including standardized questionnaires, anthropometric and blood pressure measurements, and blood sample tests.

According to standardized procedures, a well-trained investigator assisted participants in completing the standard questionnaire, which involved questions on demographic data, medical history, lifestyle, and other basic information. In regard to smoking habits, participants were classified into regular smokers, occasional smokers, or nonsmokers, which were defined as smoking nearly/more than one cigarette per day, smoking less than one cigarette per day or less than 7 cigarettes per week, and currently not smoking, respectively. Participants were also classified into three categories according to alcohol consumption. Regular drinkers, occasional drinkers, and nondrinkers referred to those who drank alcohol nearly/more than once a week, who drank alcohol less than once a week, and who currently did not drink, respectively.

Blood pressure was measured consecutively on the upper left arm of seated participants who had rested for at least 5 minutes. The resting pulse rate was recorded during blood pressure measurement. In the analyses, we used the mean value of three measurements. All anthropometric data were measured by well-trained investigators under standardized procedures. Height and weight were measured after the participants took off heavy outer clothes and shoes. Waist circumference was measured at the middle level between the lower rib margin and the iliac crest when participants were standing and wearing only underwear. Neck circumference was measured horizontally at the upper edge of the laryngeal prominence. During NC measurement, the participants kept their head upright with their eyes looking straight ahead.

The blood samples were collected at 8-9 am. Participants had fasted for at least 12 h overnight before blood sample collection. Participants with a history of diabetes and those without a history of diabetes underwent a 75 g oral glucose tolerant test or a 100 g steamed-bread meal test, respectively. Two hours later, nurses drew blood from participants again to test postprandial blood glucose levels.

### 2.3. Definition

BMI was defined as weight (kilograms) divided by the square of the height (meters). Diabetes was defined as fasting blood glucose (FBG) ≥ 7.0 mmol/L, postprandial blood glucose (PBG) ≥ 11.1 mmol/L, hemoglobin A1c (HbA1c) ≥ 6.5, and/or any self-reported history of diabetes. Prediabetes was defined as 6.1 mmol/L ≤ FBG < 7.0 mmol/L and/or PBG < 11.1 mmol/L. Hypertension was defined as systolic blood pressure (SBP) ≥ 140 mmHg, diastolic blood pressure (DBP) ≥ 90 mmHg, and/or any self-reported history of hypertension. Prehypertension was defined as 120 mmHg ≤ SBP < 140 mmHg and/or 80 mmHg ≤ DBP < 90 mmHg. Male participants with serum uric acid levels > 420 *μ*mol/L and female participants with serum uric acid levels > 360 *μ*mol/L were defined as having hyperuricemia. Cardiovascular events included a medical history of coronary heart disease, stroke, or myocardial infarction.

eGFR was calculated based on the Chronic Kidney Disease Epidemiology Collaboration (CKD-EPI) for “Asian origin” (Table 1) [18].

The loss of renal function (defined as eGFR ≤ 90 mL/min/1.73^2^) was the primary outcome, and decreased eGFR as a continuous variable was the secondary outcome.

### 2.4. Ethics

Before collecting the data, all participants signed a standardized written informed consent form. The research protocol was approved by the ethics committee of Chinese PLA General Hospital.

### 2.5. Statistical Analyses

The statistical analyses were performed using SPSS version 23.0 (IBM, Chicago, IL, USA). Continuous variables are expressed as the means ± standard deviations (SD) except for nonnormally distributed variables, which are presented as medians (interquartile ranges). Categorical variables are expressed as frequencies and percentages. For baseline characteristic analysis, we performed the chi-square test for categorical variables. In regard to continuous variables, we conducted a two-sample *t*-test for variables with a normal distribution and the Mann–Whitney *U* test for variables with a nonnormal distribution to compare the differences between the eGFR > 90 group and the eGFR ≤ 90 group, and we conducted one-way analysis of variance (ANOVA) for variables with a normal distribution and the Kruskal–Wallis *H* test for continuous variables with a nonnormal distribution to detect the differences among quartiles of neck circumferences.

To identify variables significantly associated with neck circumference or eGFR, the Pearson and Spearman correlation tests were conducted. Next, multivariable linear regression analysis was performed to evaluate the correlation between neck circumference and eGFR as a continuous variable. Furthermore, logistic regression analyses were utilized to explore the association between neck circumference and renal dysfunction, which was defined as eGFR ≤ 90 mL/min/1.73 m^2^. To control for potential confounding factors, multiple variables were adjusted in the logistic regression models. Model 1 was adjusted for age, BMI, and WHR; model 2 was further adjusted for education level, HbA1c, FPG, PBG, SBP, DBP, pulse, TGs, HDL, ALT, *γ*-GGT, and cardiovascular diseases; and model 3 was additionally adjusted for serum uric acid, smoking status, and alcohol consumption. Based on the diabetic status, hypertensive status, serum uric acid levels, history of cardiovascular diseases, and BMI, stratification analyses were performed to assess the relationships between neck circumference and renal dysfunction. The odds ratios (ORs) and 95% confidence intervals (95% CIs) are presented. *P* values < 0.05 were recognized as statistically significant. All statistical tests were two-sided and conducted separately by sex.

## 3. Results

### 3.1. Clinical Characteristics of the Study Population

A total of 8205 participants with an average age of 59.8 ± 7.9 years were included in this study. Among the subjects, 37.73% were male, while 62.27% were female. The prevalence of DM, hypertension, and cardiovascular events in all subjects was 31.8%, 48.5%, and 5.0%, respectively. An eGFR less than 90 mL/min/1.73 m^2^ was considered a decreased eGFR. A total of 1259 (37.90%) male individuals and 1622 (27.58%) female individuals had decreased eGFR. We found that age, SBP, DBP, pulse, HbA1c, FPG, PBG, TGs, HDL, serum uric acid, alanine transaminase (ALT), aspartate aminotransferase (AST), *γ*-glutamyl transpeptidase (*γ*-GGT), BMI, waist-to-hip ratio (WHR), NC, cardiovascular events, smoking status, drinking status, and education level were significantly different between the different eGFR groups. The demographics, anthropometric data, and metabolic indexes of participants are displayed in Table 2 and Supplementary Tables 1 and 2.

### 3.2. Neck Circumference Was Associated with Decreased eGFR

First, we screened out the confounding factors to perform an accurate evaluation of the association between neck circumference and eGFR. We found that some factors were related to both neck circumference and eGFR, and these factors were recognized as confounding factors. According to the criteria, age, blood glucose, blood pressure, pulse, TGs, HDL, serum uric acid (UA), aspartate aminotransferase (AST), *γ*-glutamyl transpeptidase (*γ*-GGT), BMI, waist-to-hip ratio (WHR), cardiovascular events, smoking status, drinking status, and education level were included as confounding factors (Table 3).

We first evaluated the relationship between neck circumference and eGFR as continuous variables. The results revealed that neck circumference was significantly negatively associated with the eGFR in both sexes after adjusting for all confounding factors (*β* = −0.33, 95% CI [-0.50, -0.16] in women; *β* = −0.29, 95% [-0.53, -0.06] in men) (Table 4). Then, the female and male subjects were divided into 4 groups based on the quartiles of neck circumference. The univariate logistic analysis also showed that the risk of decreased eGFR increased progressively from the first to the fourth quartiles of neck circumferences (Table 5). In model 1, after adjustment for age, BMI, and WHR, the association strengthened in male subjects and remained in female subjects. Then, further adjustment for a variety of confounding factors was performed in model 2, and the result showed that the association between NC and decreased eGFR remained robust in both sexes (Q1 as a reference; Q2: OR = 1.19, 95% CI [1.00-1.42]; Q3: OR = 1.39, 95% CI [1.10-1.75]; and Q4: OR = 1.48, 95% CI [1.17-1.86] in women; Q2: OR = 1.31, 95% CI [1.04-1.64]; Q3: OR = 1.47, 95% CI [1.13-1.90]; and Q4: OR = 1.81, 95% CI [1.31-2.51] in men). When all confounding factors were adjusted, the fourth quartile (OR = 1.32, 95% CI [1.04-1.68]) of female subjects and the third quartile (OR = 1.40, 95% CI [1.07-1.83]) and fourth quartile (OR = 1.71, 95% CI [1.22-2.38]) of male subjects still tended to have a higher risk of decreased eGFR.

### 3.3. Stratified Analyses for Different Levels of Blood Glucose, Blood Pressure, BMI, Serum Uric Acid, and History of Cardiovascular Diseases

Stratified analyses were conducted in the various subgroups (Table 6). Among the participants with prediabetes, subjects in the fourth quartile had a higher risk of decreased eGFR (Q1 as a reference; OR = 1.81, 95% CI [1.13, 2.88] in women; OR = 2.26, 95% CI [1.12, 4.57] in men). However, there were no significant relationships in either the normal population or the diabetic population. In regard to blood pressure, among the male subjects with prehypertension, subjects in the first NC quartile were taken as a reference and subjects in the fourth quartile of NC had a higher risk of decreased eGFR (OR = 2.05, 95% CI [1.06, 3.96] in men). The relationship also existed when the third (OR = 1.51, 95% CI [1.06, 2.16]) and fourth (OR = 1.57, 95% CI [1.02, 2.42]) NC quartiles were compared with the lowest quartiles of NC among male subjects with hypertension, whereas the positive relationship disappeared among female subjects. In the BMI subgroup, there was a progressively increased OR from the lowest to the highest quartiles of NC among male subjects with a BMI ≥ 24 (Q1 as a reference; Q2: OR = 1.62, 95% CI [1.14-2.30]; Q3: OR = 1.79, 95% CI [1.26-2.54]; and Q4: OR = 2.09, 95% CI [1.43-3.04]), while the association was insignificant in female subjects. The results did not differ substantially for subjects with a BMI < 24. Serum uric acid was also a critical confounding factor between NC and renal function. There was a significant association between NC and eGFR in male subjects (Q1 as a reference; Q2: OR = 1.33, 95% CI [1.04-1.70]; Q3: OR = 1.50, 95% CI [1.13-1.99]; and Q4: OR = 1.64, 95% CI [1.15-2.35]) in the normal serum uric acid group but not in the hyperuricemia group. Similar results were observed in female subjects. Additionally, among male subjects without cardiovascular diseases, the association was significant (Q1 as a reference; Q2: OR = 1.35, 95% CI [1.07-1.71]; Q3: OR = 1.47, 95% CI [1.12-1.92]; and Q4: OR = 1.72, 95% CI [1.22-2.42]). Notably, among the male participants with cardiovascular diseases, subjects in the fourth quartile had a remarkably higher risk of decreased eGFR, with an OR (95% CI) of 3.94 (1.04-14.99). In female subjects, the association only persisted in those without cardiovascular diseases.

## 4. Discussion

The onset of chronic kidney disease is often insidious and easily overlooked. A considerable proportion of CKD patients are already in an advanced stage when CKD is diagnosed. Therefore, early detection of renal function decline is of great significance. Our research suggested that high neck circumference was an important indicator of decreased kidney function. In this study, we demonstrated that higher neck circumference was significantly associated with the risk of decreased eGFR. Additionally, this association persisted after adjusting for other anthropometric parameters, traditional indicators of CKD, and lifestyle risk factors. Further stratification revealed that people with prediabetes or normal serum uric acid levels had higher risks of a decreased eGFR. When the subjects were stratified according to cardiovascular diseases, among people without cardiovascular diseases, the association persisted, while among those with cardiovascular diseases, the association only remained robust in males. Stratified analyses also showed that among male individuals, the association persisted in the subgroup of abnormal blood pressure and was strengthened in the overweight/obese subgroup. To the best of our knowledge, this study is the first large sample study about the relationship between neck circumference and decreased eGFR, which suggests renal dysfunction, in a general Chinese population.

Increasing evidence has shown that obesity is an important risk factor for renal dysfunction [19, 20]. de Boer et al. found that BMI, WC, and fat mass were each indicators of rapid eGFR loss after adjustments for age, sex, race, and smoking [21]. Neck circumference, a reliable anthropometric index for upper-body subcutaneous adiposity, has been proposed as a novel indicator of visceral obesity in recent years [3, 4]. However, research on the association between neck circumference and the risk of decreased eGFR is limited and inconsistent. One cross-sectional study of 177 high-cardiovascular-risk patients showed that higher NC is positively associated with the 24-hour urine creatinine clearance rate and microalbuminuria, and interestingly also positively correlated with eGFR [16], which may be attributed to greater survival benefits of patients with CVD who had obesity and high eGFR [22]. However, another study conducted by Yoon et al. included 2268 overweight subjects and showed that NC was significantly and inversely correlated with eGFR in male subjects, while the relationship was less prominent in female subjects [23]. The discrepancies between the abovementioned studies may be due to the differences in population characteristics, sample size, choice of eGFR equation, and confounding factors adjusted in the analyses. However, no studies have examined the correlation between neck circumference and eGFR in the general population. Our results showed for the first time that NC was significantly and negatively associated with eGFR among the general population. Similar to the finding of the study performed by Yoon et al., the association was more prominent among male subjects than among female subjects. Additionally, unlike the research performed by Liu et al. [16], we found that among individuals with cardiovascular diseases, higher NC remained positively associated with the risk of decreased eGFR. The inconsistence may result from differences in the study population, statistical methods, sample size, confounding factors adjusted in analysis, and eGFR formula. Notably, previous studies failed to adjust serum uric acid, a well-established confounding factor between obesity and CKD [9, 24, 25]. Compared with the above studies, the number of participants in our study was much larger, thereby allowing careful control of a wide spectrum of potential confounding factors, including serum uric acid, increasing the validity of our results.

It is widely accepted that visceral adipose tissue plays the main role in the effect of obesity on renal function [26]. Visceral adipose tissue, which is metabolically active, is the critical source of proinflammatory factors such as resistin and tumor necrosis factor alpha [26, 27], thereby exerting a direct renal pathogenic effect. In addition, excessive visceral adipose accumulation is a crucial cause of CKD-inducing diseases such as diabetes [28], hypertension [19], and hyperuricemia [24]. Neck circumference has been confirmed as a reliable marker of visceral fat tissue in the Chinese population [3], which may partially explain the association between neck circumference and renal dysfunction. However, after adjusting for various metabolic parameters, including WHR, one of the most widely used markers for visceral adipose tissue, the association between neck circumference and the risk of a decreased eGFR was still significant. This suggested that neck circumference had a direct impact on renal function independent of its relationship with visceral adipose tissue. The possible explanations are as follows.

NC is a valid biomarker of the subcutaneous fat of the upper body. Studies have shown that under both basal and insulin-suppressed conditions, the majority (>60%) of systemic free fatty acids (FFAs) are released from upper-body subcutaneous fat, particularly in overweight/obese people, while FFAs released from visceral adipose tissue only account for a minor proportion of the systemic FFA concentration [29–31]. Increasing evidence has shown that high levels of FFAs have renal pathogenic effects, especially on the tubulointerstitium [32], glomeruli, and podocytes [33–35]. A study by Kamijo et al. showed that FFAs accounted for severe renal tubulointerstitial inflammation and fibrosis by being reabsorbed into kidney proximal tubules [32]. Xu et al. found that saturated FFAs contributed to mitochondrial dysfunction in podocytes, leading to podocyte injury [36]. In addition, when FFAs such as palmitate and oleic acid were bound to albumin, they resulted in accelerated podocyte injury by enhancing macropinocytosis and stimulating G protein-coupled receptor signaling in podocytes [34, 36, 37]. Furthermore, FFAs have also been shown to promote endothelial dysfunction and induce systemic inflammation, both of which are pathophysiological mechanisms that may cause kidney diseases [35].

Another likely pathway underlying the association between NC and renal dysfunction is sleep apnea syndrome, which refers to intermittent, periodic airflow cessations or a decrease in airflow during sleep [38]. NC was found to positively correlate with sleep apnea syndrome [39], as increased fat deposition in the neck was a major contributor to sleep apnea syndrome [36, 38]. Studies have shown that sleep apnea syndrome is an independent risk factor for diabetes [40], hypertension [41], and cardiovascular events [42]. Additionally, increasing evidence indicates that sleep apnea syndrome is a risk factor for renal dysfunction. Two recent studies showed an independent association between snoring and CKD after adjustment for confounding factors, including metabolic diseases [43, 44]. The possible mechanisms included renal hypoxia and increased oxidative stress caused by sleep apnea directly [38]. Moreover, the possible mechanisms for the association of sleep apnea and CKD also include that individuals with sleep apnea had higher risk of microvascular abnormalities and atrial fibrillation. Lin et al. found that sleep apnea was a risk factor for retinal microvascular abnormalities in the American population [45], and a recent large cohort study showed that sleep apnea was a risk factor for a series of microvascular diseases including CKD in patients with type 2 diabetes [46]. Additionally, there were researches that indicated that individuals with sleep apnea had higher risk for atrial fibrillation [47, 48], which may lead to renal dysfunction by thromboembolism and a decline in cardiac function [49]. In our study, we investigated whether the participants snored in the last 12 months, and we found that the association between snoring and eGFR was not significant (data not shown). As sleep apnea could not be diagnosed solely based on snoring, we need further investigation on sleep apnea syndrome to better understand these issues.

Our results demonstrated that the strength of the association between NC and decreased eGFR was stronger in males than in females, which was consistent with the results from Yoon et al.'s study. One possible explanation was the difference in lipid metabolism between men and women. As mentioned above, NC was considered to be a valid index of subcutaneous fat of the upper body. Studies have revealed that the release of FFAs from upper-body subcutaneous adipose tissue in postprandial conditions is more prominent in men than in women [50], and excessive FFAs contribute to kidney injury by increasing oxidative stress and renal inflammation [32, 33]. In addition, another possible explanation was the difference in fat deposition near the upper airway between men and women. Due to the large volume of fat impinging on the pharynx, men are more vulnerable to sleep apnea syndrome [51].

Importantly, unlike previous studies, we conducted stratified analyses to identify populations in which a high NC will significantly increase the risk of decreased renal function. In stratified analyses, we found that the relationship between NC and the risk of a decreased eGFR was strengthened among people with normal serum uric acid levels but became insignificant among those with hyperuricemia. A study based on the REACTION study identified NC as a valid marker of hyperuricemia, a well-established risk factor for CKD [9]. Therefore, it was likely that the effect of hyperuricemia on eGFR covered the association between NC and eGFR. In regard to blood glucose and blood pressure, people with borderline values of blood glucose or blood pressure were more likely to have a decreased eGFR when NC was in the highest quartile, but the relationship weakened or disappeared among those with diabetes or hypertension. Epidemiological data have identified T2DM and hypertension as the major causes of renal dysfunction in China [2], so it is reasonable to speculate that the strong relationships of blood glucose and blood pressure with renal function probably weaken the relationship between NC and the risk of a decreased eGFR. Additionally, it was noteworthy that in the cardiovascular disease subgroup, male subjects at the fourth quartile of NC had a remarkably higher risk of decreased eGFR, indicating that patients with cardiovascular diseases and high NC should be alert to renal dysfunction. Moreover, we found that the association was strengthened in male subjects who were overweight/obese, which was probably due to more FFAs being released from subcutaneous adipose tissue in overweight/obese male individuals [29, 31] than in lean male individuals. Another possible mechanism was that overweight/obese individuals were with higher risk of lipid abnormalities including high levels of low-density lipoprotein, total cholesterol, total triglyceride, and low levels of high-density lipoprotein, which might have a bidirectional relationship with systemic inflammation and increase the burden of cardiovascular diseases, contributing to the progress of CKD [52, 53]. In conclusion, our results suggested that among male individuals whose NCs were in higher quartiles, those with prediabetes, prehypertension/hypertension, normal serum uric acid levels, and overweight/obesity and those with or without cardiovascular diseases were at increased risk of decreased kidney function, while among female individuals, those with prediabetes or normal serum uric acid levels and those without cardiovascular diseases were at increased risk of decreased kidney function. Therefore, we recommend that these individuals undergo regular kidney function tests to detect potential abnormal kidney function, which is conducive to the prevention and treatment of CKD.

Our study benefited from the large population and comprehensive examination, which allowed for adjustments of a variety of important covariates and stratified analyses to fully explore the relationship between NC and the decreased eGFR. However, there were some limitations to this study. First, as a cross-sectional study, it only established an association, not a causal relation. Second, the eGFR may be affected by muscle mass and metabolic status. We obtained the estimated value of GFR instead of the accurate value of GFR, which is detected by clearance of substances such as inulin or ^51^Cr-ethylenediaminetetraacetic acid. However, the direct measurement of GFR is time-consuming, impractical, and burdensome. Therefore, eGFR is a widely used marker of renal function in routine clinical practice. Third, the participants involved were mainly middle-aged, so the interpretation of the results in a younger population should be done prudently. Moreover, an imaging examination to directly quantify subcutaneous and perivascular fat in the neck would provide further evidence to validate our results.

## 5. Conclusion

In summary, NC is positively associated with the risk of decreased eGFR in the general population in China, and the association is stronger in males than in females. The association is clear among people with prediabetes or normal serum uric acid levels and those without cardiovascular diseases. Notably, among male subjects who were overweight/obese or with cardiovascular diseases, the relationship was strengthened. These results indicate that NC, as a simple and reliable anthropometric index, may contribute to risk assessment for renal dysfunction. Individuals in high NC quartiles should be aware of the risk of CKD and receive close monitoring of renal function.

## Figures and Tables

**Table 1:** The formula for the estimated glomerular filtration rate.

Sex	Serum creatinine (*μ*mol/L)	Formula (mL/min/1.73 m^2^)
Female	≤61.88	151 × (Scr/61.88)^−0.328^ × (0.993)^age^
	>61.88	151 × (Scr/61.88)^−1.210^ × (0.993)^age^
Male	≤79.56	149 × (Scr/79.56)^−0.415^ × (0.993)^age^
	>79.56	149 × (Scr/79.56)^−1.210^ × (0.993)^age^

Abbreviations: Scr: serum creatinine. The unit of age is years.

**Table 2:** Characteristics of participants categorized by sex and eGFR status.

Variables	Total	Male			Female		
		eGFR > 90	eGFR ≤ 90	*P* value	eGFR > 90	eGFR ≤ 90	*P* value
*N*	8805	2063	1259		3861	1622	
Age (years)	59.75 ± 7.88	58.88 ± 6.55	65.49 ± 8.54	<0.000	56.81 ± 6.41	63.42 ± 8.21	<0.000
SBP (mmHg)	130.37 ± 16.97	131.67 ± 16.59	135.90 ± 17.27	<0.000	127.16 ± 16.19	132.09 ± 17.45	<0.000
DBP (mmHg)	76.94 ± 9.90	79.14 ± 9.97	77.64 ± 10.85	<0.000	76.17 ± 9.26	75.41 ± 10.00	0.008
Pulse (bpm)	78.45 ± 11.72	77.98 ± 12.15	76.91 ± 12.78	0.015	79.24 ± 11.09	78.39 ± 11.63	0.011
HbA1c (%)	5.90 (5.50-6.30)	5.80 (5.50-6.40)	5.90 (5.60-6.40)	0.032	5.80 (5.50-6.20)	5.90 (5.60-6.30)	<0.000
FPG (mmol/L)	5.33 (4.94-6.06)	5.46 (5.01-6.41)	5.50 (5.03-6.40)	0.636	5.25 (4.89-5.86)	5.31 (4.92-5.94)	0.016
PBG (mmol/L)	7.98 (6.44-10.69)	8.20 (6.48-11.67)	8.37 (6.56-11.19)	0.887	7.78 (6.38-10.27)	8.13 (6.54-10.53)	0.009
TC (mmol/L)	4.91 ± 1.69	4.69 ± 2.17	4.59 ± 2.03	0.202	5.07 ± 1.49	5.04 ± 0.95	0.485
TG (mmol/L)	1.32 (0.97-1.88)	1.29 (0.91-1.89)	1.27 (0.94-1.87)	<0.000	1.32 (0.98-1.84)	1.44 (1.07-1.98)	<0.000
HDL (mmol/L)	1.43 ± 0.43	1.33 ± 0.35	1.30 ± 0.33	0.057	1.51 ± 0.50	1.48 ± 0.40	0.033
LDL (mmol/L)	3.13 ± 1.43	3.00 ± 1.63	2.89 ± 1.64	0.068	3.21 ± 0.83	3.27 ± 2.00	0.250
UA (mmol/L)	304.07 ± 75.95	328.47 ± 71.50	362.06 ± 81.16	<0.000	271.49 ± 61.27	305.57 ± 69.82	<0.000
ALT (U/L)	17.20 (13.30-23.60)	18.60 (14.40-25.70)	17.40 (13.70-23.20)	<0.000	16.80 (13.00-23.10)	16.40 (12.60-21.43)	0.001
AST (U/L)	19.10 (16.40-22.50)	19.00 (16.10-22.40)	19.40 (16.50-22.50)	0.046	18.90 (16.40-22.45)	19.30 (16.90-22.93)	0.001
GGT (U/L)	20.80 (15.50-30.15)	25.30 (18.90-37.10)	24.20 (18.10-35.00)	0.011	18.50 (14.15-26.20)	18.80 (14.60-25.80)	0.414
BMI (kg/m^2^)	25.50 ± 3.53	25.46 ± 3.19	25.82 ± 3.29	0.001	25.34 ± 3.67	25.68 ± 3.79	0.001
WHR	0.89 ± 0.06	0.91 ± 0.06	0.92 ± 0.05	0.033	0.86 ± 0.06	0.87 ± 0.06	<0.000
NC (cm)	35.52 ± 3.28	38.05 ± 2.66	38.38 ± 2.64	<0.000	33.81 ± 2.47	34.16 ± 2.49	<0.000
Cardiovascular events (%)	436 (5.0%)	113 (5.5%)	109 (8.7%)	<0.000	132 (3.4%)	82 (5.1%)	0.004
Hypertension (%)	4274 (48.5%)	1027 (49.8%)	828 (65.8%)	<0.000	1534 (39.7%)	885 (54.6%)	<0.000
Diabetes (%)	2800 (31.8%)	748 (36.3%)	449 (35.7%)	0.729	1087 (28.2%)	516 (31.8%)	0.007
Drinking status (%)				<0.000			0.341
No	6303 (71.6%)	749 (36.3%)	565 (44.9%)	0.000	3499 (90.6%)	1490 (91.9%)	
Occasional drinkers	1317 (15.0%)	586 (28.4%)	340 (27.0%)		286 (7.4%)	105 (6.5%)	
Regular drinkers	1185 (13.5%)	728 (35.3%)	354 (28.1%)		76 (2.0%)	27 (1.7%)	
Smoking status (%)				<0.000			0.863
No	7187 (81.6%)	1022 (49.5%)	821 (65.2%)		3766 (97.5%)	1578 (97.3%)	
Occasional smokers	149 (1.7%)	79 (3.8%)	35 (2.8%)		24 (0.6%)	11 (0.7%)	
Regular smokers	1469 (16.7%)	962 (46.6%)	403 (32.0%)		71 (1.8%)	33 (2.0%)	
Education				<0.000			<0.000
Illiteracy	105 (1.2%)	2 (0.1%)	7 (0.6%)		28 (0.7%)	68 (4.2%)	
Primary school	472 (5.4%)	54 (2.6%)	96 (7.6%)		163 (4.2%)	159 (9.8%)	
Junior high school	2943 (33.4%)	757 (36.7%)	424 (33.7%)		1199 (31.1%)	563 (34.7%)	
Senior high school	3869 (43.9%)	913 (44.3%)	408 (32.4%)		1940 (50.2%)	608 (37.5%)	
College	1416 (16.1%)	337 (16.3%)	324 (25.7%)		531 (13.8%)	224 (13.8%)	

Continuous data are shown as mean ± standard deviation or median (interquartile range), and categorical data are shown as frequency. Abbreviations: eGFR: estimated glomerular filtration rate; SBP: systolic blood pressure; DBP: diastolic blood pressure; HbA1c: hemoglobin A1c; FBG: fasting blood glucose; PBG: postprandial blood glucose; TC: total cholesterol; TG: triglyceride; HDL: high-density lipoprotein cholesterol; LDL: low-density lipoprotein cholesterol; UA: serum uric acid; ALT: alanine aminotransferase; AST: aspartate aminotransferase; *γ*-GGT: *γ*-glutamyl transferase; BMI: body mass index; WHR: waist-to-hip ratio; NC: neck circumference.

**Table 3:** Correlation of variables with neck circumference and eGFR stratified by sex.

Variables	Male				Female			
	Neck circumference		eGFR		Neck circumference		eGFR	
	*r*	*P* value	*r*	*P* value	*r*	*P* value	*r*	*P* value
Age (years)	-0.09	<0.000	-0.49	<0.000	0.04	0.001	-0.54	<0.000
SBP (mmHg)	0.17	<0.000	-0.14	<0.000	0.23	<0.000	-0.18	<0.000
DBP (mmHg)	0.15	<0.000	0.09	<0.000	0.16	<0.000	0.06	<0.000
Pulse (bpm)	0.02	0.352	0.06	0.001	0.03	0.037	0.03	0.017
HbA1c (%)	0.15	<0.000	-0.04	0.035	0.24	<0.000	-0.08	<0.000
FPG (mmol/L)	0.15	<0.000	0.00	0.820	0.24	<0.000	-0.04	0.006
PBG (mmol/L)	0.14	<0.000	<0.000	0.986	0.25	<0.000	-0.03	0.033
TC (mmol/L)	0.02	0.399	0.20	<0.000	-0.03	0.017	0.00	0.809
TG (mmol/L)	0.28	<0.000	0.01	0.509	0.28	<0.000	-0.08	<0.000
HDL (mmol/L)	-0.31	<0.000	0.04	0.018	-0.25	<0.000	0.03	0.047
LDL (mmol/L)	0.01	0.433	0.05	0.003	0.02	0.155	-0.01	0.426
UA (mmol/L)	0.20	<0.000	-0.25	<0.000	0.29	<0.000	-0.29	<0.000
ALT (U/L)	0.18	<0.000	0.11	<0.000	0.20	<0.000	0.06	<0.000
AST (U/L)	0.01	0.672	-0.04	0.017	0.03	0.067	-0.07	<0.000
GGT (U/L)	0.25	<0.000	0.11	<0.000	0.26	<0.000	0.00	0.865
BMI (kg/m^2^)	0.73	<0.000	-0.06	0.002	0.69	<0.000	-0.03	0.014
WC (cm)	0.73	<0.000	-0.07	<0.000	0.69	<0.000	-0.10	<0.000
Cardiovascular events (%)	0.05	0.003	-0.09	<0.000	0.06	<0.000	-0.06	<0.000
Hypertension (%)	0.22	<0.000	-0.16	<0.000	0.23	<0.000	-0.17	<0.000
Diabetes (%)	0.12	<0.000	0.01	0.482	0.18	<0.000	-0.03	0.027
Drinking status (%)	0.04	0.014	0.12	<0.000	0.03	0.024	0.04	0.006
Smoking status (%)	0.02	0.319	0.20	<0.000	0.04	0.003	0.01	0.620
Education (%)	0.02	0.254	0.01	0.481	-0.10	<0.000	0.19	<0.000

Abbreviations: eGFR: estimated glomerular filtration rate; OR: odds ratio; CI: confidence interval; BMI: body mass index; SBP: systolic blood pressure; DBP: diastolic blood pressure; HbA1c: hemoglobin A1c; FBG: fasting blood glucose; PBG: postprandial blood glucose; ALT: alanine transaminase; *γ*-GGT: *γ*-glutamyl transpeptidase; TG: triglyceride; HDL: high-density lipoprotein cholesterol.

**Table 4:** Multiple linear regression analyses of association between neck circumference and eGFR.

	Model 1		Model 2		Model 3	
	*β* (95% CI)	*P* value	*β* (95% CI)	*P* value	*β* (95% CI)	*P* value
Total	-0.42 (-0.57, -0.28)	<0.000	-0.45 (-0.59, -0.31)	<0.000	-0.35 (-0.48, -0.21)	<0.000
Female	-0.40 (-0.57, -0.22)	<0.000	-0.46 (-0.63, -0.28)	<0.000	-0.33 (-0.50, -0.16)	<0.000
Male	-0.37 (-0.62, -0.13)	0.003	-0.37 (-0.62, -0.13)	0.003	-0.29 (-0.53, -0.06)	0.014

Model 1: adjusted for age, BMI, and WHR. Model 2: additionally adjusted for SBP, DBP, pulse, HbA1c, FPG, PBG, TG, HDL, ALT, *γ*-GGT, and cardiovascular disease. Model 3: additionally adjusted for serum uric acid, smoking, and drinking. Abbreviations: eGFR: estimated glomerular filtration rate; OR: odds ratio; CI: confidence interval; BMI: body mass index; WHR: waist-to-hip ratio; SBP: systolic blood pressure; DBP: diastolic blood pressure; HbA1c: hemoglobin A1c; FBG: fasting blood glucose; PBG: postprandial blood glucose; ALT: alanine transaminase; *γ*-GGT: *γ*-glutamyl transpeptidase; TG: triglyceride; HDL: high-density lipoprotein cholesterol.

**Table 5:** Logistic regression models evaluating the association of neck circumference with the risk of decreased eGFR.

	Q1	Q2	*P* value	Q3	*P* value	Q4	*P* value
		OR (95% CI)		OR (95% CI)		OR (95% CI)	
Female							
Unadjusted	1.00 (reference)	1.20 (1.03-1.39)	0.021	1.42 (1.17-1.72)	<0.000	1.49 (1.27-1.75)	<0.000
Model 1	1.00 (reference)	1.19 (1.00-1.41)	0.055	1.41 (1.12-1.78)	0.004	1.42 (1.13-1.78)	0.002
Model 2	1.00 (reference)	1.19 (1.00-1.42)	0.055	1.39 (1.10-1.75)	0.006	1.48 (1.17-1.86)	0.001
Model 3	1.00 (reference)	1.14 (0.95-1.37)	0.153	1.31 (1.03-1.66)	0.030	1.32 (1.04-1.68)	0.021
Male							
Unadjusted	1.00 (reference)	1.22 (1.01-1.48)	0.040	1.30 (1.07-1.58)	0.009	1.50 (1.21-1.87)	<0.000
Model 1	1.00 (reference)	1.32 (1.06-1.65)	0.014	1.47 (1.14-1.90)	0.003	1.75 (1.27-2.40)	0.001
Model 2	1.00 (reference)	1.31 (1.04-1.64)	0.020	1.47 (1.13-1.90)	0.004	1.81 (1.31-2.51)	<0.000
Model 3	1.00 (reference)	1.26 (0.99-1.59)	0.056	1.40 (1.07-1.83)	0.013	1.71 (1.22-2.38)	0.002

Model 1: adjusted for age, BMI, and WHR. Model 2: additionally adjusted for education, SBP, DBP, pulse, HbA1c, FPG, PBG, TG, HDL, ALT, *γ*-GGT, and cardiovascular disease. Model 3: additionally adjusted for serum uric acid, smoking, and drinking. Abbreviations: eGFR: estimated glomerular filtration rate; OR: odds ratio; CI: confidence interval; BMI: body mass index; WHR: waist-to-hip ratio; SBP: systolic blood pressure; DBP: diastolic blood pressure; HbA1c: hemoglobin A1c; FBG: fasting blood glucose; PBG: postprandial blood glucose; ALT: alanine transaminase; *γ*-GGT: *γ*-glutamyl transpeptidase; TG: triglyceride; HDL: high-density lipoprotein cholesterol.

**Table 6:** The association between neck circumference and the risk of decreased eGFR in subgroups.

Variable	Q1	Q2	*P* value	Q3	*P* value	Q4	*P* value
		OR (95% CI)		OR (95% CI)		OR (95% CI)	
Female							
Blood glucose^a^							
Normal	1.00 (reference)	1.17 (0.90-1.50)	0.238	1.49 (1.04-2.13)	0.029	1.18 (0.82-1.69)	0.380
Prediabetes	1.00 (reference)	1.30 (0.89-1.89)	0.170	1.43 (0.89-2.29)	0.142	1.81 (1.13-2.88)	0.013
Diabetes	1.00 (reference)	0.99 (0.68-1.43)	0.944	1.01 (0.63-1.63)	0.963	1.09 (0.69-1.71)	0.721
Blood pressure^b^							
Normal	1.00 (reference)	1.17 (0.82-1.68)	0.383	1.41 (0.83-2.40)	0.208	1.42 (0.83-2.46)	0.203
Prehypertension	1.00 (reference)	1.21 (0.86-1.70)	0.285	1.45 (0.93-2.26)	0.105	1.30 (0.83-2.02)	0.253
Hypertension	1.00 (reference)	1.15 (0.87-1.52)	0.317	1.27 (0.89-1.80)	0.183	1.33 (0.94-1.86)	0.104
BMI^c^							
BMI < 24	1.00 (reference)	1.06 (0.83-1.37)	0.637	1.25 (0.75-2.09)	0.392	1.31 (0.80-2.17)	0.288
BMI ≥ 24	1.00 (reference)	1.18 (0.89-1.57)	0.251	1.28 (0.93-1.75)	0.126	1.22 (0.91-1.64)	0.181
Serum uric acid^d^							
Uric acid ≤ 360	1.00 (reference)	1.21 (1.00-1.46)	0.049	1.58 (1.23-2.03)	<0.000	1.36 (1.06-1.75)	0.017
Uric acid > 360	1.00 (reference)	1.03 (0.57-1.85)	0.928	0.72 (0.35-1.46)	0.357	1.68 (0.86-3.28)	0.129
Cardiovascular disease^e^							
No	1.00 (reference)	1.18 (0.99-1.42)	0.065	1.38 (1.08-1.75)	0.009	1.42 (1.12-1.80)	0.003
Yes	1.00 (reference)	1.28 (0.44-3.70)	0.650	1.55 (0.44-5.46)	0.500	2.92 (0.81-10.58)	0.103
Male							
Blood glucose^a^							
Normal	1.00 (reference)	1.24 (0.88-1.75)	0.229	1.32 (0.87-2.01)	0.186	1.45 (0.84-2.48)	0.181
Prediabetes	1.00 (reference)	1.05 (0.64-1.72)	0.844	1.22 (0.70-2.14)	0.488	2.26 (1.12-4.57)	0.024
Diabetes	1.00 (reference)	1.37 (0.88-2.12)	0.160	1.58 (0.99-2.53)	0.056	1.81 (1.02-3.20)	0.041
Blood pressure^b^							
Normal	1.00 (reference)	1.29 (0.71-2.32)	0.400	1.36 (0.63-2.91)	0.433	1.67 (0.58-4.78)	0.340
Prehypertension	1.00 (reference)	1.44 (0.93-2.24)	0.103	1.09 (0.65-1.83)	0.755	2.05 (1.06-3.96)	0.034
Hypertension	1.00 (reference)	1.15 (0.83-1.60)	0.399	1.51 (1.06-2.16)	0.024	1.57 (1.02-2.42)	0.043
BMI^c^							
BMI < 24	1.00 (reference)	1.01 (0.71-1.43)	0.978	0.89 (0.51-1.58)	0.696	1.26 (0.21-7.60)	0.804
BMI ≥ 24	1.00 (reference)	1.62 (1.14-2.30)	0.008	1.79 (1.26-2.54)	0.001	2.09 (1.43-3.04)	<0.000
Serum uric acid^d^							
Uric acid ≤ 420	1.00 (reference)	1.33 (1.04-1.70)	0.023	1.50 (1.13-1.99)	0.005	1.64 (1.15-2.35)	0.007
Uric acid > 420	1.00 (reference)	1.05 (0.51-2.15)	0.895	1.34 (0.62-2.89)	0.461	2.31 (0.93-5.77)	0.073
Cardiovascular disease^e^							
No	1.00 (reference)	1.35 (1.07-1.71)	0.013	1.47 (1.12-1.92)	0.006	1.72 (1.22-2.42)	0.002
Yes	1.00 (reference)	0.79 (0.29-2.10)	0.630	1.21 (0.42-3.46)	0.728	3.94 (1.04-14.99)	0.044

Model a: adjusted for age, BMI, WHR, SBP, DBP, pulse, TG, HDL, ALT, *γ*-GGT, education level, cardiovascular disease, serum uric acid, smoking, and drinking. Model b: adjusted for age, WHR, BMI, HbA1c, FPG, PBG, pulse, TG, HDL, ALT, *γ*-GGT, education level, cardiovascular disease, serum uric acid, smoking, and drinking. Model c: adjusted for age, WHR, SBP, DBP, pulse, HbA1c, FPG, PBG, TG, HDL, ALT, *γ*-GGT, education level, cardiovascular disease, serum uric acid, smoking, and drinking. Model d: adjusted for age, WHR, BMI, SBP, DBP, pulse, HbA1c, FPG, PBG, TG, HDL, ALT, *γ*-GGT, education level, cardiovascular disease, smoking, and drinking. Model e: adjusted for age, WHR, BMI, SBP, DBP, pulse, HbA1c, FPG, PBG, TG, HDL, ALT, *γ*-GGT, education level, serum uric acid, smoking, and drinking. Abbreviations: eGFR: estimated glomerular filtration rate; OR: odds ratio; CI: confidence interval; BMI: body mass index; WHR: waist-to-hip ratio; SBP: systolic blood pressure; DBP: diastolic blood pressure; HbA1c: hemoglobin A1c; FBG: fasting blood glucose; PBG: postprandial blood glucose; ALT: alanine transaminase; *γ*-GGT: *γ*-glutamyl transpeptidase; TG: triglyceride; HDL: high-density lipoprotein cholesterol.

## Data Availability

The datasets used and/or analysed during this study are not freely available for the sake of participants' privacy protection but are available from the corresponding author on reasonable request.

## Supplementary Materials

Supplementary Materials Supplementary Table 1: characteristics of male participants categorized by neck circumference. Supplementary Table 2: characteristics of female participants categorized by neck circumference.
