# Supplementary material for: Association between Neck Circumference and the Risk of Decreased Estimated Glomerular Filtration Rate in the General Population of China: A Cross-Sectional Study
Source: Biomed Res Int. 2020 Nov 23;2020:3496328. doi: 10.1155/2020/3496328 (PMC7707937; doi:10.1155/2020/3496328)
Supplement: Supplementary Materials — Supplementary Table 1: characteristics of male participants categorized by neck circumference. Supplementary Table 2: characteristics of female participants categorized by neck circumference. [file 3496328.f1.docx]

Supplementary table 1. Characteristic of male participants categorized by neck circumference.

| **Variables** | **Total** | **Q1（≤36cm）** | **Q2（36-38cm）** | **Q3（38-40cm）** | **Q4（>40cm）** | ***P value*** |
| --- | --- | --- | --- | --- | --- | --- |
| N | 3322 | 880 | 988 | 880 | 574 |  |
| Age(years) | 61.4±8 | 62.2±8 | 61.8±8 | 60.8±7.9 | 60.3±8.3 | <0.000 |
| SBP(mmHg) | 133.3±17 | 129±17 | 133.3±17.1 | 135.3±16.5 | 136.6±16.1 | <0.000 |
| DBP(mmHg) | 78.6±10.3 | 76.1±10.1 | 78.7±10.1 | 79.7±10.4 | 80.4±10.2 | <0.000 |
| Pulse(bpm) | 77.6±12.4 | 77.4±12.3 | 77.4±12.7 | 77.2±12 | 78.6±12.6 | 0.175 |
| HbA1c(%) | 5.9(5.5-6.4) | 5.7(5.4-6.2) | 5.8(5.5-6.4) | 5.9(5.6-6.5) | 6(5.6-6.7) | <0.000 |
| FPG(mmol/L) | 5.5(5-6.4) | 5.3(4.9-5.9) | 5.5(5-6.4) | 5.5(5.1-6.5) | 5.7(5.1-7.1) | <0.000 |
| PBG(mmol/L) | 8.3(6.5-11.5) | 7.6(6.2-10.2) | 8.2(6.4-11.6) | 8.7(6.8-11.9) | 9.1(7-12.3) | <0.000 |
| TC(mmol/L) | 4.7±2.1 | 4.6±0.9 | 4.7±2 | 4.6±1.7 | 4.7±3.7 | 0.666 |
| TG(mmol/L) | 1.3(0.9-1.9) | 1.1(0.8-1.5) | 1.3(0.9-1.8) | 1.4(1-2.1) | 1.5(1.1-2.2) | <0.000 |
| HDL(mmol/L) | 1.3±0.3 | 1.5±0.4 | 1.3±0.3 | 1.3±0.3 | 1.2±0.3 | <0.000 |
| LDL(mmol/L) | 3±1.6 | 2.9±0.8 | 3±2.4 | 3±1.6 | 2.9±0.8 | 0.135 |
| UA(mmol/L) | 341.2±77 | 320.7±67.6 | 339.5±77.5 | 350.3±76.4 | 361.6±83 | <0.000 |
| ALT(U/L) | 18.1(14-24.8) | 16.5(12.8-21.9) | 17.9(13.9-24.3) | 19.3(15.2-25.8) | 20.3(15.2-28.3) | <0.000 |
| AST(U/L) | 19.2(16.3-22.4) | 19.1(16.3-22.5) | 19.2(16.3-22) | 19.2(16.3-22.6) | 19.2(16.4-23.1) | 0.548 |
| GGT(U/L) | 25(18.6-36.3) | 20.9(16.1-30.7) | 24.5(18.4-35) | 27.1(20.6-38.1) | 30.4(22.4-43.4) | <0.000 |
| BMI（Kg/m^2^） | 25.6±3.2 | 22.6±2.2 | 25.1±2.3 | 26.8±2.2 | 29.1±2.8 | <0.000 |
| WHR | 0.92±0.05 | 0.88±0.05 | 0.91±0.05 | 0.93±0.05 | 0.95±0.05 | <0.000 |
| eGFR（mL/min/1.73^2^） | 92.5±15 | 93.8±15.2 | 92.3±13.9 | 92±14.4 | 91.5±17.2 | 0.017 |
| eGFR (mL/min/1.732) |  |  |  |  |  | 0.002 |
| >90 | 2063(62.1%) | 587(66.7%) | 614(62.1%) | 534(60.7%) | 328(57.1%) |  |
| ≤90 | 1259(37.9%) | 293(33.3%) | 374(37.9%) | 346(39.3%) | 246(42.9%) |  |
| Cardiovascular events(%) | 222(6.7%) | 39(4.4%) | 75(7.6%) | 59(6.7%) | 49(8.5%) | 0.009 |
| Hypertension(%) | 1855(55.8%) | 344(39.1%) | 563(57%) | 549(62.4%) | 399(69.5%) | <0.000 |
| Diabetes(%) | 1197(36%) | 246(28%) | 347(35.1%) | 347(39.4%) | 257(44.8%) | <0.000 |
| Drinking status(%) |  |  |  |  |  | 0.044 |
| No | 1314(39.6%) | 360(40.9%) | 410(41.5%) | 346(39.3%) | 198(34.5%) |  |
| Occasional drinkers | 926(27.9%) | 247(28.1%) | 264(26.7%) | 229(26%) | 186(32.4%) |  |
| Regular drinkers | 1082(32.6%) | 273(31%) | 314(31.8%) | 305(34.7%) | 190(33.1%) |  |
| Smoking status(%) |  |  |  |  |  | 0.941 |
| No | 1843(55.5%) | 496(56.4%) | 546(55.3%) | 488(55.5%) | 313(54.5%) |  |
| Occasional smokers | 114(3.4%) | 33(3.8%) | 36(3.6%) | 28(3.2%) | 17(3%) |  |
| Regular smokers | 1365(41.1%) | 351(39.9%) | 406(41.1%) | 364(41.4%) | 244(42.5%) |  |
| Education（%） |  |  |  |  |  | 0.363 |
| Illiteracy | 9(0.3%) | 2(0.2%) | 3(0.3%) | 2(0.2%) | 2(0.3%) |  |
| Primary school | 150(4.5%) | 45(5.1%) | 52(5.3%) | 29(3.3%) | 24(4.2%) |  |
| Junior high school | 1181(35.6%) | 329(37.4%) | 343(34.7%) | 294(33.4%) | 215(37.5%) |  |
| Senior high school | 1321(39.8%) | 325(36.9%) | 398(40.3%) | 380(43.2%) | 218(38%) |  |
| College | 661(19.9%) | 179(20.3%) | 192(19.4%) | 175(19.9%) | 115(20%) |  |
| Continuous data are shown as mean standard deviation or median (interquartile range) and categorical data are shown as frequency (%). | | | | | | |
|  |  |  |  |  |  |  |
| Abbreviations: eGFR, estimated glomerular filtration rate; SBP, systolic blood pressure; DBP, diastolic blood pressure; HbA1c: hemoglobin A1c; FBG, fasting blood glucose; PBG, postprandial blood glucose; TC, total cholesterol; TG, triglyceride; HDL, high-density lipoprotein cholesterol; LDL, low-density lipoprotein cholesterol; UA, serum uric acid; ALT, alanine aminotransferase; AST, aspartate aminotransferase; γ-GGT, γ-glutamyl transferase; BMI, body mass index; WHR: waist to hip ratio; NC, neck circumference. | | | | | | |
|  |  |  |  |  |  |  |
|  |  |  |  |  |  |  |
|  |  |  |  |  |  |  |

Supplementary table 2. Characteristic of female participants categorized by neck circumference.

| **Variables** | **Total** | **Q1（≤32cm）** | **Q2（32-34cm）** | **Q3（34-35cm）** | **Q4（>35cm）** | ***P value*** |
| --- | --- | --- | --- | --- | --- | --- |
| N | 5483 | 1592 | 1837 | 711 | 1343 |  |
| Age(years) | 58.8±7.6 | 58.3±7.5 | 58.6±7.6 | 59.1±7.8 | 59.3±7.7 | 0.003 |
| SBP(mmHg) | 128.6±16.7 | 123.6±16.2 | 128.4±16.6 | 130.3±15.9 | 133.9±16.3 | <0.000 |
| DBP(mmHg) | 75.9±9.5 | 73.7±8.9 | 76.2±9.5 | 77±9.1 | 77.6±9.8 | <0.000 |
| Pulse(bpm) | 79±11.3 | 78.8±11.4 | 78.4±11 | 79.1±10.5 | 79.9±11.7 | 0.004 |
| HbA1c(%) | 5.8(5.5-6.3) | 5.7(5.4-6) | 5.8(5.5-6.2) | 5.9(5.6-6.4) | 6(5.7-6.6) | <0.000 |
| FPG(mmol/L) | 5.3(4.9-5.9) | 5.1(4.8-5.5) | 5.3(4.9-5.9) | 5.3(5-6) | 5.5(5.1-6.4) | <0.000 |
| PBG(mmol/L) | 7.9(6.4-10.4) | 7.1(5.9-8.9) | 7.8(6.4-10.3) | 8.1(6.7-10.4) | 8.9(7.2-11.7) | <0.000 |
| TC(mmol/L) | 5.1±1.3 | 5.1±1.6 | 5.1±1.5 | 5±1 | 5±1 | 0.17 |
| TG(mmol/L) | 1.4(1-1.9) | 1.1(0.9-1.6) | 1.4(1-1.9) | 1.5(1.1-2.1) | 1.6(1.2-2.2) | <0.000 |
| HDL(mmol/L) | 1.5±0.5 | 1.7±0.6 | 1.5±0.4 | 1.4±0.3 | 1.4±0.3 | <0.000 |
| LDL(mmol/L) | 3.2±1.3 | 3.2±0.8 | 3.2±0.9 | 3.3±1.8 | 3.3±1.8 | 0.113 |
| UA(mmol/L) | 281.6±65.8 | 257.6±58.6 | 279.3±62 | 291.3±65 | 307.9±68.4 | <0.000 |
| ALT(U/L) | 16.7(12.9-22.7) | 15.1(11.9-19.8) | 16.4(12.9-22.1) | 17.3(13.3-23.5) | 18.9(14.3-26.9) | <0.000 |
| AST(U/L) | 19(16.5-22.6) | 19(16.5-22.4) | 19(16.4-22.3) | 19(16.4-22.3) | 19.2(16.6-23.7) | 0.002 |
| GGT(U/L) | 18.6(14.3-26.1) | 16.2(12.8-22.4) | 18.1(14.1-25.4) | 19.7(15.5-26.6) | 21.9(16.5-30.9) | <0.000 |
| BMI（Kg/m^2^） | 25.4±3.7 | 22.4±2.5 | 25.1±2.5 | 26.7±2.7 | 28.8±3.6 | <0.000 |
| WHR | 0.87±0.06 | 0.83±0.06 | 0.87±0.06 | 0.88±0.05 | 0.90±0.06 | <0.000 |
| eGFR（mL/min/1.73^2^） | 95.7±13.8 | 96.8±13.1 | 95.9±13.4 | 95.2±13.2 | 94.2±15.3 | <0.000 |
| eGFR (mL/min/1.732) |  |  |  |  |  | <0.000 |
| >90 | 2063(62.1%) | 587(66.7%) | 614(62.1%) | 534(60.7%) | 328(57.1%) |  |
| ≤90 | 1259(37.9%) | 293(33.3%) | 374(37.9%) | 346(39.3%) | 246(42.9%) |  |
| Cardiovascular events(%) | 214(3.9%) | 34(2.1%) | 71(3.9%) | 40(5.6%) | 69(5.1%) | <0.000 |
| Hypertension(%) | 2419(44.1%) | 490(30.8%) | 789(43%) | 338(47.5%) | 802(59.7%) | <0.000 |
| Diabetes(%) | 1603(29.2%) | 303(19%) | 539(29.3%) | 216(30.4%) | 545(40.6%) | <0.000 |
| Drinking status(%) |  |  |  |  |  | 0.214 |
| No | 4989(91%) | 1472(92.5%) | 1661(90.4%) | 647(91%) | 1209(90%) |  |
| Occasional drinkers | 391(7.1%) | 93(5.8%) | 145(7.9%) | 50(7%) | 103(7.7%) |  |
| Regular drinkers | 103(1.9%) | 27(1.7%) | 31(1.7%) | 14(2%) | 31(2.3%) |  |
| Smoking status(%) |  |  |  |  |  | 0.042 |
| No | 5344(97.5%) | 1565(98.3%) | 1790(97.4%) | 696(97.9%) | 1293(96.3%) |  |
| Occasional smokers | 35(0.6%) | 6(0.4%) | 13(0.7%) | 4(0.6%) | 12(0.9%) |  |
| Regular smokers | 104(1.9%) | 21(1.3%) | 34(1.9%) | 11(1.5%) | 38(2.8%) |  |
| Education（%） |  |  |  |  |  | <0.000 |
| Illiteracy | 96(1.8%) | 26(1.6%) | 26(1.4%) | 16(2.3%) | 28(2.1%) |  |
| Primary school | 322(5.9%) | 66(4.1%) | 108(5.9%) | 58(8.2%) | 90(6.7%) |  |
| Junior high school | 1762(32.1%) | 452(28.4%) | 607(33%) | 218(30.7%) | 485(36.1%) |  |
| Senior high school | 2548(46.5%) | 756(47.5%) | 857(46.7%) | 321(45.1%) | 614(45.7%) |  |
| College | 755(13.8%) | 292(18.3%) | 239(13%) | 98(13.8%) | 126(9.4%) |  |
| Continuous data are shown as mean standard deviation or median (interquartile range) and categorical data are shown as frequency (%). | | | | | | |
|  |  |  |  |  |  |  |
| Abbreviations: eGFR, estimated glomerular filtration rate; SBP, systolic blood pressure; DBP, diastolic blood pressure; HbA1c: hemoglobin A1c; FBG, fasting blood glucose; PBG, postprandial blood glucose; TC, total cholesterol; TG, triglyceride; HDL, high-density lipoprotein cholesterol; LDL, low-density lipoprotein cholesterol; UA, serum uric acid; ALT, alanine aminotransferase; AST, aspartate aminotransferase; γ-GGT, γ-glutamyl transferase; BMI, body mass index; WHR: waist to hip ratio; NC, neck circumference. | | | | | | |
|  |  |  |  |  |  |  |
|  |  |  |  |  |  |  |
|  |  |  |  |  |  |  |
